# Inferring animal social networks and leadership: applications for passive monitoring arrays

**DOI:** 10.1098/rsif.2016.0676

**Published:** 2016-11

**Authors:** David M. P. Jacoby, Yannis P. Papastamatiou, Robin Freeman

**Affiliations:** 1 Institute of Zoology, Zoological Society of London, Regent's Park, London NW1 4RY, UK; 2 Department of Biological Sciences, Florida International University, 3000 N.E. 151st Street, North Miami, FL 33181, USA

**Keywords:** animal tracking, Bayesian inference, shark sociality, social structure, telemetry

## Abstract

Analyses of animal social networks have frequently benefited from techniques derived from other disciplines. Recently, machine learning algorithms have been adopted to infer social associations from time-series data gathered using remote, telemetry systems situated at provisioning sites. We adapt and modify existing inference methods to reveal the underlying social structure of wide-ranging marine predators moving through spatial arrays of passive acoustic receivers. From six months of tracking data for grey reef sharks (*Carcharhinus amblyrhynchos*) at Palmyra atoll in the Pacific Ocean, we demonstrate that some individuals emerge as leaders within the population and that this behavioural coordination is predicted by both sex and the duration of co-occurrences between conspecifics. In doing so, we provide the first evidence of long-term, spatially extensive social processes in wild sharks. To achieve these results, we interrogate simulated and real tracking data with the explicit purpose of drawing attention to the key considerations in the use and interpretation of inference methods and their impact on resultant social structure. We provide a modified translation of the GMMEvents method for R, including new analyses quantifying the directionality and duration of social events with the aim of encouraging the careful use of these methods more widely in less tractable social animal systems but where passive telemetry is already widespread.

## 1. Introduction

Developments in biologging techniques are facilitating novel ways in which information on animal social behaviours and movements are gathered, substantially increasing the quantity, quality and longevity of interactions that can be monitored simultaneously [1–3]. Proximity-based social networks (PBSNs), for example, offer a means to reconstruct social structure in intractable species from the frequency of paired spatial associations between tracked individuals [4]. Recent analyses, designed to extract social networks from automated spatiotemporal time series [5,6], have utilized telemetry data to infer social networks for hundreds of individuals over periods of years having significant impact on our understanding of the evolutionary processes driving population dynamics [7–9], enabling researchers to measure social structure over vast sampling areas. Such methods, to date, have adopted Bayesian inference, specifically Gaussian mixture modelling (GMM) approaches, originally developed as pattern recognition tools in machine learning, with the explicit aim of tackling the non-trivial issue of how best to sample and construct graphs of social association from automated telemetry data.

This particular application of Bayesian inference has arisen, in part, due to the logistical limitations of deploying devices and retrieving data from spatial proximity loggers that directly record animal contact rates (e.g. [10]). Logistical constraints still limit broad swaths of ecological systems from the large-scale deployment of such technology, particularly in systems where the tagged animals are unlikely to be encountered again. For example, despite a few interesting studies demonstrating proof of concept [11,12], proximity logging of social animals in the marine environment remains underdeveloped relative to terrestrial systems where their use has enabled hugely detailed, automated mapping of social networks *in situ* and in real time [2,10,13]. Fundamentally, the marine environment rules out the use of radio telemetry where many of the developments in this field have occurred (e.g. [14,15]). The logistical constraints to data storage, retrieval and battery life of such systems in marine environments or the ability to deploy and collect sufficient numbers of proximity loggers to capture the social structure of wide-ranging animals remains prohibitive and hence the emergence of analytical inference methods that can help to fill this gap with no extra risk or cost associated [16].

Typically, in systems where directed interactions are not obvious or easily recorded, there is an implicit assumption that individuals in close spatial proximity are associating with one another, a concept known as the ‘gambit of the group’, which often fails to distinguish social and spatial processes (as discussed in [4,17]). Constructing social networks from temporal *and* spatial data addresses this to an extent, but also provides a solution to the often subjective assignment of aggregation time windows (i.e. sampling periods) to time-series data [5,18]. Thus, an automated approach explores the inherent structure already present in the visitation profile of tagged animals, detecting the most likely ‘clustering events’, of variable size, that reflect the variation expected in dynamic animal societies [19]. These methods rely on individual- and group-level patterns in the arrival of animals to specific areas of interest (e.g. feeding stations). But how ‘clustered’ do the data actually need to be to infer a biologically meaningful signal? And can such methods be used, for example, to sample the underlying social structure of a community of free-ranging animals where the data-stream is comparatively sparse owing to natural fission–fusion within the population. The data that have typically been analysed to date using this method (i.e. birds at feeding stations), often already contain obvious structure in the visitation profile—imposed either by experimental manipulation and/or by known circadian rhythm, sensor on/off patterns and prior knowledge of foraging that occurs in groups (e.g. [7,20]). In other systems, such natural structure in the data may not always be known and therefore cannot necessarily be assumed.

The methodology behind GMM inference of animal social structure and how this mitigates the potential bias associated with choosing a sampling period is already discussed in depth by Psorakis and colleagues [5,6]. Equally, there are a growing number of studies using this same model system (i.e. RFID-tagged birds of the family Paridae in Wytham Woods Oxfordshire, UK) to demonstrate the real potential of these analyses to explore broad questions in population and evolutionary ecology (e.g. [7,21]). Consequently, we do not discuss either in great detail here. Rather we aim to explore the broader utility of such methods for capturing the underlying social structure of animals moving through, but not necessarily attracted to, arrays of spatial receivers, where data are typically rather sparse and sporadic, and may have no natural ‘breaks’ in the pattern of detections. Recently, Armansin and colleagues used a small acoustic array to demonstrate that if receivers are placed close enough together to overlap, hyperbolic positioning can be used to reconstruct the social associations of site-attached, benthic elasmobranchs, using an approach akin to PBSN construction [22]. Building on such work and using time-series data gathered by a spatially extensive, fixed array of non-overlapping acoustic receivers at a remote Pacific Atoll, we address the following questions: (i) can a GMM approach reconstruct social associations in simulated structured data? (ii) How are social network metrics influenced by data partitioning? (iii) Can leadership behaviour be inferred from within-event detection chronology and social duration? We translate a modified version of the GMMEvents code from [5] into open R code and highlight a series of considerations for those wishing to construct social networks on the long-term associations of animals from passive tracking or logging data.

## 2. Material and methods

### 2.1. The structure of sparsely distributed biologging data

A wealth of automated instrumentation now exists to record individual animals tagged with both passive and active electronic tracking devices [3,23]. Biologging, and in particular, the tracking of animals in autonomous fixed-receiver arrays (AFAs), has long been employed to enhance our understanding of animal space use, visitation patterns and residency behaviour for a broad range of marine and terrestrial species [24,25] and with clear application in species conservation [26,27] and movement ecology [28]. In some instances, the data recovered from arrays of receivers can be relatively sparse temporally as animals enter and leave the area under surveillance, sometimes for long periods.

To illustrate the application of these inference methods to such data, we use underwater, acoustic tracking data on the movements of sharks through an AFA of hydrophone receivers. The data used in this study were gathered as part of a long-term field study into the movements of reef-associated predators at Palmyra Atoll (5°53′ N, 162°05′ W) in the central Pacific Ocean. Specifically, we analyse data on the movements of grey reef sharks (*Carcharhinus amblyrhynchos*, Bleeker 1856), tagged with long-life V16 acoustic transmitters (*n* = 44), monitored within an array of 63 VR2 W hydrophone receivers (VEMCO, Halifax, Nova Scotia), over a period of 3 years (552 026 detections). Sharks were tagged, as part of a larger study, to quantify patterns of space use of predators at an island scale Marine Protected Area. Detection ranges of acoustic receivers can vary greatly depending on the local environment and typically range testing of receiver arrays to determine detection probabilities is not always adequately considered within study designs [29]. For the current data, a subsample of receivers (owing to time constraints in such a remote location) in three common habitats were range-tested revealing average detection ranges of 500 m on the forereef, 250 m on the backreef and 350 m in lagoon habitats. For the purposes of this study, we work to the backreef range as a minimum; however, we acknowledge that future research addressing specific ecological hypotheses will require an added weighting to the social network inference whereby those receivers with smaller detection ranges contribute more to the social network than those with very large detection ranges.

**Figure 1.**
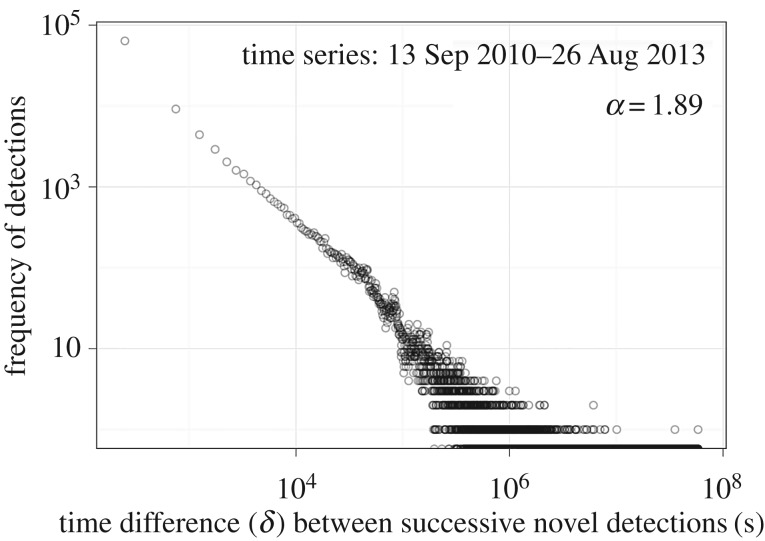
Difference in individual arrival times across receivers. The heavy-tailed, log–log frequency distribution of the complete shark detection time differences across the full 3 years, demonstrating that sparse visitation data can still be clustered temporally and, therefore, applicable for a GMM approach.

These telemetry data were deemed representative of a system, unperturbed by experimenter influence, where individual sharks enter and leave the array, and where sporadic gathering events occur between multiple individuals, across multiple locations in a dynamic, fission–fusion manner. To address the applicability of the GMM approach for acoustic tracking, the underlying structure of the data was explored by plotting the frequency distribution of time differences, *δ*(*t_z_*) = *t_z_* – *t_z_*_−1_, between the arrival of consecutive individuals (*z*) at receivers across the array, ignoring repeated detections by the same individual. Even among temporally sparse detection data, we observe the characteristic ‘heavy-tailed’ distribution (*α* = 1.9) representing high numbers of short time intervals, interspersed with low numbers of long intervals (figure 1) that is indicative of *clustered* aggregation data [5].

### 2.2. Modification of the GMMEvents code

The modified R code for the GMMEvents analyses, simulated and real trial datasets, along with user instructions are available at https://github.com/JacobyD/gmmevents. This code, which is reliant upon a Gaussian mixture model with variational Bayesian inference (VBGMM), enables users to load a data-stream containing *time*, *individual* and *location* from one or many logging devices and extract the following: (i) the number of clustering events at each location and collectively, as determined by the GMM; (ii) the mean event times (centroids) for each event including other summary statistics, and (iii) five *N × N* adjacency matrices, *A* = [*a_ij_*], defined as a square, individual-by-individual matrix quantifying different aspects of the paired (hereafter referred to as dyadic) co-occurrences. These include matrices (i) and (ii) which are count matrices (‘AdjMat_count’ and ‘AdjMat_pre_sig_count’) that provide an accumulated count of the within-event detections for the individual in a dyad that was present for the shortest period, 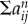 , as per [5], both pre- and post-significance test; (iii) a prime matrix that is an accumulation of binary scores across events (AdjMat_PRIME) representing the number of separate events dyads were detected together. Of course, these matrices will be correlated, as one is derived from the other, so why not simply use the count matrix as in previous studies? We argue that the best indication of social behaviour from relatively course-scale telemetry data is the number of times individuals co-occur across different events, but that this relationship can be refined further by considering the duration or mean duration of those co-occurrences (see Inferring directionality and duration of social ties); (iv) we also construct a directed, asymmetric version of the PRIME matrix (AdjMat_PRIME_dir) and (v) a duration matrix (AdjMat_dur) pertaining to the accumulated time in seconds that dyadic detections overlap across all events they co-occur in. All adjacency matrices are discussed in context below.

### 2.3. Simulated data trial

**Figure 2.**
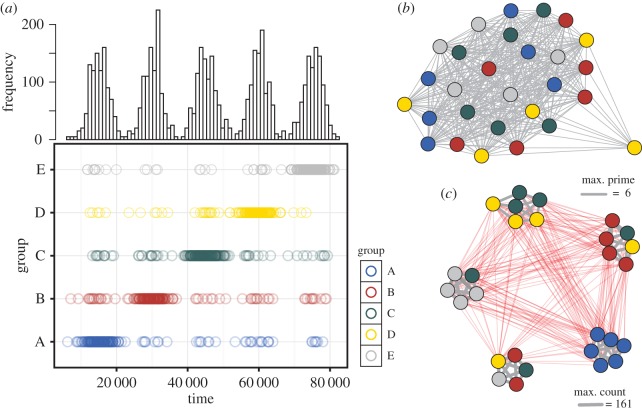
Simulated animal detection data. A time series of ‘detections’ is colour coded by groups of individuals with detections occurring across five centres of mass (s.d. = 0.025) at a single location. Individuals (*n* = 28) are each detected 800 times, with the probability of individuals within each group (A–E) being assigned 90% of the time to one cluster (*a*). Networks of significant, binary co-occurrence across the five events identified by the VBGMM (prime—*b*) and the accumulated minimum count of detections between overlapping individuals across those events (count—*c*), where red edges reflect non-significant co-occurrences.

Given that the data appear to suggest aggregated activity at the receivers, we examined the efficacy of the Gaussian mixture model (GMM) approach to capture the underlying social structure of shark populations. The first step in this process was to see how well the model performed with highly structured, simulated data with ‘forced’ social affiliations. To do this, we simulated 4000 random occurrence times (*t*), with a known distribution of *K* mean event times before randomly apportioning individual detections (*i*) to those known *K*, with varying degrees of probability and associated noise. The simulated data were designed to represent a time series where each individual is likely to be found in one of five events more than any other, creating aggregations in time with a given probability of 0.9, and where the participants are known in advance (figure 2*a*). We then tested whether these *a priori* known social affiliations were detectable with the GMM approach.

### 2.4. Partitioning the data-stream

As GMMs can be computationally expensive and time-consuming, data are often divided into portions, run separately and then combined, post model, into one adjacency matrix representing the broad social structure within the population. For some social systems, discrete breaks in the visitation profile might occur naturally or be assumed in advance; for example, foraging behaviour in mixed-species population of tits (Paridae) is known to occur only during daylight hours [21], providing an obvious division of the data. Social behaviour in other systems might be much less defined temporally and consequently, we explore the influence of data-stream division on the eventual outcome of the inferred social structure.

We take a week-long data-stream, equivalent to our simulation, of 4000 detections of grey reef sharks and analyse it in its entirety before splitting it into two equal-sized portions of 2000 each and re-running the analysis on each portion, combining the results. As the GMM is not deterministic, we then iterate this process 50 times to produce error estimates for our comparisons. While 50 times is a relatively low number of permutations, the GMM is extremely time-consuming and computationally demanding, involving multiple permutation tests of its own and so this number of iterations was deemed sufficient and manageable to assess error in comparing the split and complete data-streams. The model output and resultant social network characteristics were explored using Wilcoxon's signed-rank test and Spearman's rank test.

### 2.5. Inferring directionality and duration of social ties

The idea that there is an inherent structure within the time series, but also further structure pertaining to relative dyadic detection profiles within each identified clustering event, allows us to extend the methodology outlined in [5], providing new insight into the nature of social events inferred from telemetry data. For each event, we extract the chronology of arrival times at locations between individuals to infer directionality of social affiliations and identify followership and leadership behaviours. In essence, this relies on identifying leadership based on dyadic interactions. It is important to stress here that we measure leadership in order to assess individual influence within the population in the context of social network position, not to understand dominance or social hierarchies; additional data at a finer spatial scale would be required to achieve this goal. Leadership, however, need not necessarily be reliant on complex social information transfer between individuals. Studies on human groups in controlled conditions, for example, demonstrate that without any prior knowledge about group mates, individuals were able to identify and benefit from individual leader behaviour [30]. We acknowledge that there are more sophisticated techniques that measure leadership in coordinated group activities, such as the leadership inference framework outlined in [31]; however, we believe such approaches are more suitable in the context of collective behavioural processes where the mechanistic foundations of collective behaviour are being tested directly. We also extract co-occurrence duration by retrieving the number of overlapping detections between dyads within events and their associated times.

Even semi-gregarious species that demonstrate a fission–fusion approach to social behaviour require a mechanism for spatiotemporal coordination often resulting in the emergence of leadership and followership behaviour within a population [32]. To illustrate the utility of the extensions we have added to the code, we extract inference on leader–follower behaviour for a six-month period of the grey reef shark acoustic tracking data (approx. 125 000 detections). Leadership scores (*L_i_*) were constructed for each shark based on the proportion of each individual's degree that was represented by *in degree* (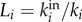) within the directional adjacency matrix, dependent upon whether individuals within a dyad were detected first or second within an event. We then explored the predictability of *L_i_* from individual network attributes of sex, total length (TL) and mean event duration (fixed effects), as well as individual (random effect), using a linear mixed-effects model (‘nlme’ package in R). Mean durations were calculated as the mean time individuals co-occurred within an event across all dyads present, which was then logged to achieve normality. All analyses throughout were carried out in R v. 3.2.3.

## 3. Results

### 3.1. Capturing the structure of simulated data

The simulation showed that the GMM could successfully identify the five distinct events and the distribution of individuals across those clustering events (figure 2). The prime matrix (see Modification of the GMMEvents code (2.2)) suggests a highly homogeneous network (figure 2*b*) reflecting the fact that some individuals from all groups (A–E) were detected in all five events within the simulated single location. Conversely, the ‘count’ network that represents the minimum number of detections per significant dyad provides an indication of those dyads exhibiting some longevity in their co-occurrences, and shows five distinct social groups (once non-significant, red edges were removed) with some variability in membership as a result of the noise introduced into the simulated data (figure 2*c*). A sparse and noisy signal is indicative of the nature of tracking data and the timescales over which these large animals roam in and out of trackable areas. Hence, construction of social networks for wide-ranging species from telemetry data requires many receiver locations and ideally long periods of tag retention time, as per our shark tracking data-stream where individuals were tagged internally.

### 3.2. Influence of data-stream partitioning

**Figure 3.**
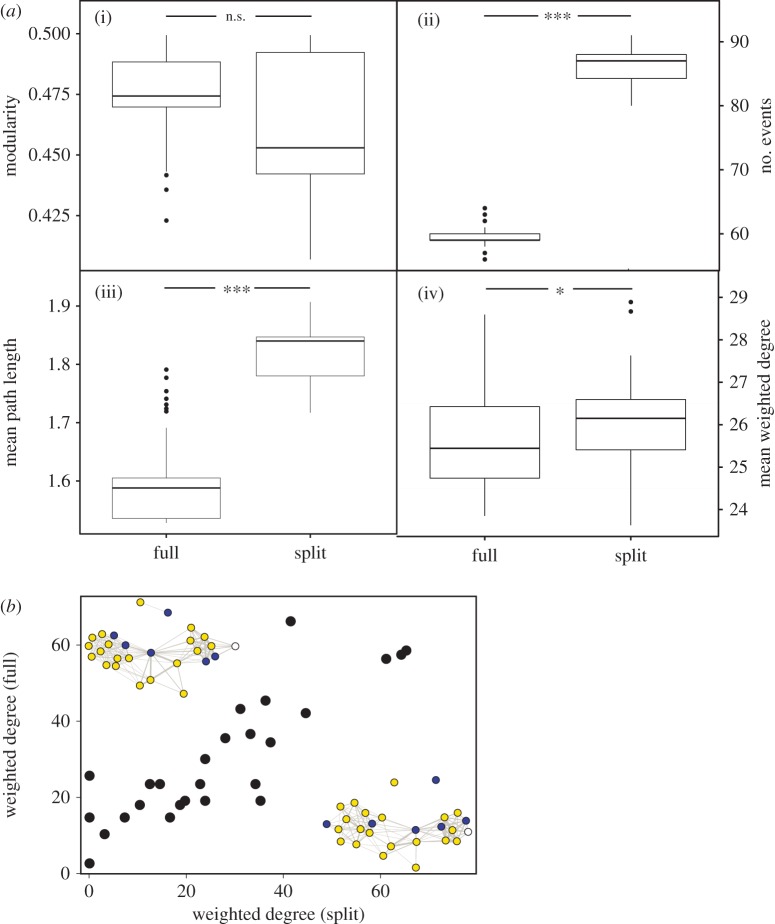
Comparison of social network structure from split and complete data-streams. The weighted social network for 27 individual sharks over the period of one week constructed from combining data split into two portions of 2000 lines (split) each and the same data run through the model in ‘full’ (yellow = female, blue = male, white = unknown). Encouragingly, there are strong similarities in community structure measured by modularity (*a*(i)) and a significant correlation (Spearman's rank, *r* = 0.89, *p* < 0.001) in individual weighted degree (*k_i_*)(*b*); however, differences in the number of events (*a*(ii)) identified by the VBGMM can influence subtle network properties, such as the average path length (*L*) or global measures of degree that can impact flow and diffusion within the network (*a*(iii) and (iv)). Significance = *p < 0.05, **p < 0.01, ***p < 0.001.

We demonstrate that when splitting the data-stream, the GMM reports a significantly higher number of clustering events (*V* = 0, *p* < 0.001, figure 3*a*(ii)) than when the model is run on the full data-stream. Despite this, there were significantly similar structural properties at the individual level with weighted individual degree (*k_i_*) showing a strong positive correlation (Spearman's rank, *r* = 0.89, *p* < 0.001, figure 3*b*) and similarities in community structure, both visually and statistically (*V* = 819, *p* > 0.05, figure 3*a*(i),*b*). We do, however, identify differences in the average path length (*V* = 0, *p* < 0.001, figure 3*a*(iii)) and the overall mean degree (*V* = 339.5, *p* < 0.05, figure 3*a*(iv)) even within this relatively small-scale network.

Variation in the global structural properties of a network might not necessarily have an immediate impact on a given individual (although it can), but it can substantially alter the dynamics of flow within a system and this has clear implications for information and disease transmission rates across the network [33,34]. Indeed, the long-range links in a network (i.e. increased average path length) can be linked to the emergence of more virulent infectious diseases as the cost to the pathogen of wiping out a local host population is reduced by this increased reach [35]. Therefore, the ability of the model to capture the true global properties of the network can be critical in situations where pathogens require a tipping point to persist [36], or where information dissemination across the group is reliant on a threshold or quorum of individuals obtaining and sharing it [37,38]. It is worth being mindful of the potential risk of failing to capture such thresholds from the structural properties of inferred networks where the data have been divided prior to processing. Therefore, despite the computational costs involved, we encourage practitioners to think carefully about when and how to partition data for analytical purposes, using where possible, ecologically determined divisions that represent natural breaks in aggregation behaviour.

### 3.3. Inferring directionality and duration of social ties

**Figure 4.**
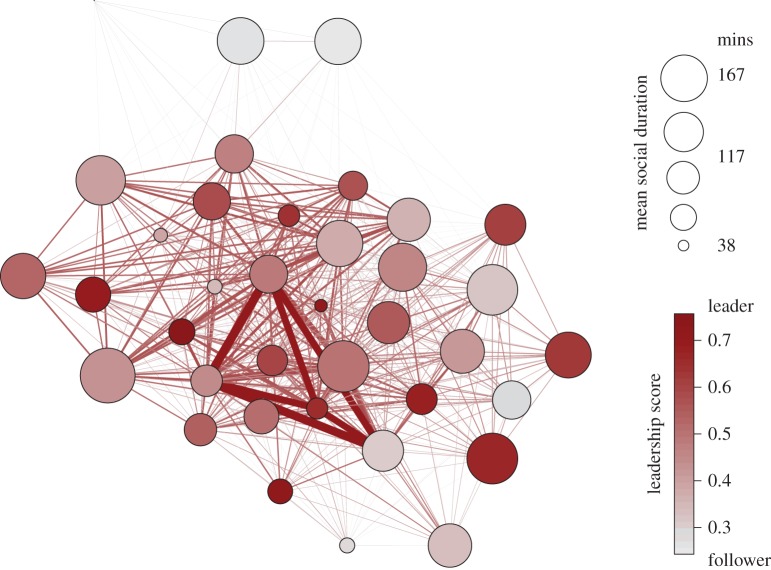
Leadership behaviour within the social network. The inferred social interactions of 37 individual grey reef sharks at Palmyra Atoll, over a period of six months in 2013. Node colour represents a follower (light grey) to leader (dark red) scale and node size demonstrates the mean duration of an individual's co-occurrence with all other conspecifics. The weight and colour of network edges show the strength of social affiliations based on the number of times individuals co-occurred in separate events together.

Across sharks of a limited size range (owing to the constraints associated with minimum tagging size), there was considerable variation in leadership tendencies and mean social duration (figure 4). Typically, the strongest social ties occurred between individuals of intermediate *L* score (i.e. those that tended to lead and follow in equal measures) and interestingly, these individuals had longer social durations than conspecifics with a propensity to lead. This is intuitive as *natural leaders* will likely move on to a new location before *natural followers*. Unlike TL, sex and mean social duration were both significant predictors of leadership, as was the interaction between sex and TL (*p* < 0.05). The emergent structure within the network appeared to suggest that male sharks were less likely to lead, as were those individuals that maintained longer co-occurrences with conspecifics (results summarized in table 1). This analysis demonstrates leadership and its ecological predictors; it is important to acknowledge, however, that social affiliations are highly dynamic and often context-dependent and that temporal analysis would be required to understand the stability of leadership behaviours through time and across context [39].

**Table 1.** Sources of variation contributing to the emergence of leadership within a shark population. The results are from the final linear mixed-effects model fit by RMEL which included an interaction term between sex and total length (TL) but not between either of these with log-transformed mean duration per event (MDPE) (all fixed effects) and including a random effect of individuals. The bold values are significant at the *p* < 0.05 level.

fixed effects	model coefficients	s.e.	*t*_d.f._	*p-*value
sex	−1.015	0.460	−2.205_29_	**0**.**0355**
TL	−0.001	0.001	−0.709_29_	0.4840
log_10_(MDPE)	−0.077	0.032	−2.405_29_	**0**.**0228**
sex : TL	0.007	0.003	2.178_29_	**0**.**0377**

## 4. Discussion

Grey reef sharks can display a range of behaviour, from central place refuging with site residency, to more nomadic wider ranging movements and evidence of social associations between individuals [40,41]. In some locations, grey reef sharks also form all female aggregations in very shallow water, likely related to gestation [42]. The social dynamics of these aggregations, however, have yet to be explored, in part, due to the challenges associated with developing an unbiased strategy for sampling the social network. Knowledge of the fine-scale structural dynamics of shark aggregations, and their temporal stability is important in both an ecological and conservation context [43] and can be used, for example, to understand reproductive strategies or for highlighting periods of population vulnerability [44].

To this end, we explore the utility of GMMs for retrieving inference on social network structure from telemetry data of spatiotemporal co-occurrences, modifying the currently available methodologies to extract additional behavioural information on the timing and directionality of dyadic interactions. We demonstrate leadership patterns in a shark population over a six-month period and explain some of the predictors of these patterns. Our ability to demonstrate sex as a significant driver of leadership is tantalizing and indicative of the importance of fine-scale intersexual interactions in structuring shark population dynamics [43]. By exploring leadership over such a long period, however, we are likely missing some of the nuances associated with individual- and group-level behaviours, in addition to the role of abiotic factors in determining shifts in the timing of such behaviours. Reproduction, for example, will no doubt be seasonal as individual grey reef sharks tend not to show year-round fidelity to a single reef area [40], but also spatially explicit as mating, gestation and pupping perhaps all occur in specific (and potentially very different) localities [45]. Perhaps most interestingly, these methods might help to shed light on the complex interplay between individual home ranges, contact rates and the potential asymmetry between conspecifics regarding localized information about resource distribution.

### 4.1. Significance testing and edge weighting

The GMMEvents package has a number of randomization procedures incorporated. Firstly, the significance test assigns the probabilities of individual detections to a particular distribution or clustering event (this is irrespective of individual ID). Secondly, and more crucially from a social network perspective, these data are then randomized at the bipartite graph stage—that is an individual to event network—which means that the detection *frequency* of an individual within an event can be constrained. This is significant in the light of recent evidence arguing that to avoid bias, null models for hypothesis testing in animal social networks should rely on randomizations of the raw data-stream over node-based randomizations (see [46,47] for discussion), especially where logging devices have large detection ranges such as the acoustic receivers in this study. Tagged animals can, for example, rest within the range of a receiver and record many successive detections providing added structure within the data-stream and also an opportunity to use this within-event structure to weight our associations. Crucially, permutation of the bipartite graph allows us to control for the gregarious nature of individuals and the greater probability of association between individuals with overlapping home ranges, while randomizing the fact that individuals might have social preferences within those aggregations. Network studies in captivity, where individuals have been observed, often control for individual gregariousness (e.g. [48]) or group size structuring (e.g. [49]), but it is less obvious how best to do this when working with abstract time-series data from biologging devices. Spiegel *et al*. [4] suggest that randomization procedures that adopt data-stream permutation still fail to fully discriminate between the spatial heterogeneity and social attraction, arguing that movement tracks be randomized within individuals not between. Spiegel and colleagues' promising methodology offers an eloquent solution to this problem, although for wide-ranging, fission–fusion species, there remains the rather subjective challenge of assigning a time window to randomize within an individual's movement track.

### 4.2. Confidence in our inferences

Having explored the utility of the GMM approach for sparse, fission–fusion detection data, it is prudent to discuss a number of important considerations for how we interpret the inferences we draw from such analyses. A reasonable question to pose would be how much confidence do we have in the sensitivity of our sensors from which we infer social behaviour? For example, in this study, we work with an approximate receiver detection range of 250 m (radius), suggesting that a co-occurrence can be recorded between individuals up to approximately 500 m apart. We noted earlier that the effective detection range can be highly variable and is not always fully accounted for in many studies [29,50], suggesting further refinement of network weightings is needed as these methods develop. The current methodology proposed in this paper remains unvalidated and fine-scale, contact network data, such as that obtained from proximity loggers, are much needed to confirm the accuracy with which these techniques truly capture social associations in marine organisms [2,51]. Terrestrial studies have taught us that the reliability with which proximity networks capture social processes can be highly variable [52] and can influence how we interpret network structure [53]. Despite the scope for uncertainty here also, we argue that if dyads are continually detected in different clustering events and at different locations, then our confidence in a social association is likely to increase proportionally. Combining this with our ability to further weight or rank edges based on the duration of shared detections, we can improve our confidence still further. Caveats aside, there is clear merit in these methodologies given that the longevity of social network data achieved using conventional passive tracking/logging in combination with GMM analyses and the ability to track hundreds of individuals simultaneously [5,8,21] continue to far exceed even the most sophisticated proximity logging systems currently available.

Although seemingly stating the obvious, one important consideration that has clear bearing on the structural properties of the network is the degree of temporal overlap between tags. One can imagine using the GMM approach to infer a network that indicates social segregation between two subgroups, something that appears to be a statistically significant effect when examined for assortment; such a result, however, might materialize from minimal temporal overlap of the individuals in each group. Put simply, if there is little overlap between the last detections of one group and the first detections of a newly tagged group, yet the data are all included in the GMM analysis, then we are likely to return a false-positive result, a type I error. Rarely are all individuals tagged at the beginning of a study and so researchers interested in understanding the social structure of a population using this method must balance the scientific questions (e.g. how does social behaviour change with season?), with utilizing data with the greatest overlap of individuals (i.e. a section of the time-series with the highest number of tagged animals at liberty).

Finally, we are confident that this method can correctly identify statistically significant dyadic partnerships within an event, controlling for, to a degree, spatial bias in individual home ranges. However, the nature of the data collection prohibits us from confirming whether the method always captures a biological event *per se*. Thus, inference methods do not necessarily always address whether the assignment of a clustering event in time is indicative of an aggregation of biological significance in our target species. For this reason, validation of the results with visual or recorded observations is much needed.

### 4.3. Conclusion

For better or worse, a limited number of successful and highly refined technological systems dominate the global telemetry market. This, however, has encouraged broad collaboration and the coordinated deployment of thousands of tracking devices across diverse taxa, all gathering standardized data [54]. While developments in proximity logging have been limited in its ability to measure the social structure of marine organisms, inference methods offer the opportunity to interrogate the growing list of long-term, time-series telemetry data (e.g. the Ocean tracking Network: http://oceantrackingnetwork.org/) with new questions. We discuss some of the strengths and constraints of this methodology and provide code containing a number of modifications to explore the drivers of behaviour from telemetry data. In doing so, we construct the first long-term leadership network in free-ranging sharks demonstrating how such tools will be crucial in helping to elucidate the ecological role and conservation implications of sociality and behavioural hierarchies in marine ecosystems [23].
